# Editorial: Advances in sports science: latest findings and new scientific proposals, volume III

**DOI:** 10.3389/fpsyg.2026.1858846

**Published:** 2026-05-13

**Authors:** Rubén Maneiro, Mario Amatria, José Luís Losada, Gudberg K. Jonsson, Antonio Ardá, Iyán Iván-Baragaño

**Affiliations:** 1 Department of Special Didactics, Faculty of Education and Sports Sciences, University of Vigo, Vigo, Spain; 2 Department of Physical Education and Sport, Pontifical University of Salamanca, Salamanca, Spain; 3 Department of Social Psychology and Quantitative Psychology, University of Barcelona, Barcelona, Spain; 4 Social Science Research Institute and Human Behavior Laboratory, University of Iceland, Reykjavík, Iceland; 5 Department of Physical and Sport Education, University of A Coruña, A Coruña, Spain; 6 Department of Physical Education and Sport, Faculty of Physical Activity and Sport Sciences, University of León, León, Spain

**Keywords:** collective sports, high performance, individual sports, match analysis, performance analysis

In recent decades, sport sciences have evolved into a markedly multidisciplinary field, integrating physiological, psychological, technical-tactical, and methodological approaches to better understand performance and participation in sport contexts. The present Research Topic brings together 26 contributions that reflect this complexity, offering empirical evidence and systematic reviews that advance knowledge from complementary perspectives.

A first group of studies focuses on psychological processes, wellbeing, and social dynamics in sport. Zhu and Wen examined the effects of a basketball-based intervention on positive body image among female college students, demonstrating that improvements are mediated by both embodiment experiences and self-compassion, which operate independently and sequentially. Lucibello et al. explored the relationship between weight-related shame and flourishing in adolescent girls, distinguishing between current and anticipatory shame and showing that only current shame negatively impacts wellbeing. In grassroots sport, González-Hernández et al. analyzed the effects of exposure to violence, revealing that higher exposure increases aggressive behaviors while influencing prosocial tendencies. Zhang and Yu proposed the stress–depletion–aggression (SDA) model, integrating physiological, cognitive, and motivational mechanisms to explain the emergence of aggressive behavior in high-pressure football contexts. Furthermore, Zhang, Kinoshita, et al. investigated the relationship between social capital and hedonic wellbeing, reporting generally positive associations with some variability. In addition, Becan et al. analyzed the effects of music on psychological resilience, motivation, and physical performance, identifying differences according to gender, experience level, and sport type, as well as variations between pre- and post-exercise conditions.

A second block of studies addresses technical-tactical performance analysis in team sports, providing insights into decision-making and efficiency. Amatria et al. analyzed the effectiveness of pick-and-roll actions in elite women's basketball, showing that collective solutions, rapid decision-making, and specific game moments (particularly the second and third quarters) are associated with greater success. In women's volleyball, Sitti and Rungbuhe examined spiking efficiency according to attack type, court zone, and phase of play, demonstrating that certain central zones and attack conditions yield higher efficiency. Wang and Wang compared first-attack performance in elite women's volleyball teams between the 2020 and 2024 Olympic Games, identifying evolving offensive trends and structural changes in gameplay. Similarly, Zhang, Wang, et al. analyzed positional technical differences between men's and women's basketball at the Olympic level, highlighting distinct performance profiles that require position-specific training and tactical approaches.

A third group focuses on physiological, biomechanical, and neuromuscular determinants of performance, emphasizing physical adaptations and underlying biological mechanisms. Wang, Zhen, et al., through a network meta-analysis, compared strength and plyometric training, concluding that strength training is more effective for sprint performance, whereas plyometric training shows greater benefits for jump performance. Pismiri et al. analyzed affective responses and fatigue under hypoxic and normoxic conditions, demonstrating that different environmental and training protocols elicit distinct responses to variables such as energy, tension, and fatigue. Petrovic et al. examined age-related differences in jumping and sprinting performance among basketball players, finding superior results in older athletes, likely due to neuromuscular development. Ogata et al. investigated the relationship between ground reaction forces and sacrum acceleration during change-of-direction movements, identifying significant associations during specific phases of the movement. Additionally, Lu et al. explored psychomotor efficiency using electroencephalography (EEG), revealing neural activation differences between individuals with varying levels of expertise, associated with motor control and cognitive processing.

Another group of studies addresses coaching, training management, and applied sport contexts, providing insights for practitioners. Cano-Cuartero et al. analyzed swimming coaches' perceptions of training periodization and performance management, revealing a predominance of traditional approaches combined with contextual adaptations and limited athlete involvement in planning. Johnson et al. examined in-season training loads in American football, identifying clear differences between training and competition demands in variables such as distance covered, speed, and mechanical load. Ha et al. investigated safety practices in golf training environments, showing that perceived safety positively influences trust in coaches' and athletes' intention to continue participating.

The Research Topic also includes studies on perceptual-cognitive processes and decision-making in officiating. Wang, Wu, et al. analyzed visual tracking performance in sports officials, identifying differences in accuracy based on expertise and officiating roles. Complementarily, Wang, Chen, et al. examined visual search behavior in basketball referees, demonstrating that expert referees exhibit more efficient gaze patterns and higher decision-making accuracy than less experienced officials.

From a methodological and measurement perspective, Shin et al. validated the “creactability” scale in football using Rasch modeling, confirming its unidimensional structure, reliability, and construct validity. Sáez de Ocáriz et al. developed and validated the CHEAT-1 questionnaire to assess perceptions of cheating in traditional sports, demonstrating strong psychometric properties and applicability in educational and sports contexts.

Several studies adopt systematic review and evidence synthesis approaches. González-del Castillo and Barbero-Alcocer reviewed the effects of physical activity on executive functions in children, identifying improvements particularly in inhibitory control, although with heterogeneous results across studies. Bezuglov et al. reviewed the ergogenic effects of music in team sports, highlighting its positive influence on both physiological and psychological variables, despite variability in protocols. Carlone et al. examined the relationship between the gut microbiome and athletic performance, identifying emerging evidence linking microbiota composition to metabolic processes, recovery, and training adaptation.

Finally, other contributions expand the field to health-related and emerging research areas. Nie and Fan investigated the effects of different exercise modalities on inhibitory control in individuals with internet addiction, showing modality-specific neurocognitive responses and highlighting the therapeutic potential of physical activity.

This Research Topic highlights the increasing complexity and interdisciplinarity of sport sciences. The diversity of approaches, methodologies, and populations underscores the importance of integrating multiple perspectives to better understand performance, behavior, and health in sport contexts. Future research should continue to advance methodological rigor, strengthen interdisciplinary integration, and promote evidence-based practices that enhance both performance and wellbeing in sport.

